# Progress in Ophthalmology

**Published:** 1898-07-30

**Authors:** 


### PROGRESS IN OPHTHALMOLOGY.

**Argentamin in Conjunctivitis.**—Imre, of Budapest, says of Argentamin, on which we commented in our last article, that applications with a brush of a solution at normal temperature caused no hyperæmia; the greater number of patients felt absolutely no burning sensation; in some, itching continued for ten minutes, especially in those whose conjunctiva secreted abundantly. The drug produces no discoloration, as does the usual argent nit. solution. Irrigation with water after brushing on the drug is unnecessary, but it is recommended to wash off the first deposit and then paint the surface a second time; the drug is not then decomposed, and has a more energetic action. The results, he finds, are as good as those obtained with a 1-2 per cent. solution of argent. nit. The astringent action is prompt and remarkable. The painting may be repeated four or five times a day without causing too great an irritation. The remedy was employed in conjunctivitis, catarrhal, blenorrhoic, and granular, and was also well borne in corneal ulcer and iritis. Nitrate of silver, in sufficiently strong solutions to be of any service, is so irritating that its application is limited to once or twice a day; this is insufficient for disinfecting purposes. Argentamin forms no precipitate with fluids containing sodium chloride, and experiments have proved that argentamin possesses greater antiseptic powers than argent. nit., and, at the same time, passes more deeply into the tissues; he observes that argentamin was decomposed on the surface of the tissues in the same manner as argent. nit. was, but that there was never the grey deposit seen in the latter, which meant that argentamin did not enter into combination with the albumen of the tissues.

**Large Fragment of Wood in the Retrotarsal Fold.**—A peculiar case is recorded by J. W. Croskery,[2] in which a man came complaining merely of a "sore eye," and the following history was given: Eight months before, in Russia, while chopping wood, was struck in the eye by a fragment. The eye gave very little trouble, so no attention was paid to it. Six months after the accident he left Russia to go to America, and, passing through Germany, his eye was inspected, his vision tested, and all was reported to be right. On arrival in America the eye was a little irritable, but nothing out of the way, so no opinion was sought. Eventually, when he did visit the clinic the appearance was one of a purulent ophthalmia. Upon everting the upper lid a piece of wood (18 mm. long, 6 mm. wide, and 3 mm. thick) was at once discovered resting in the retrotarsal fold. The upper lid was covered with a thick tenacious membrane, and at the inner angle was a mass of granular tissue. The whole surface of the upper lid had a granulation look, the uppe and inner quadrant of the cornea was slightly hazy, but the bulbar conjunctiva was free from any hyperæmia. The wood was removed, the granulation tissue excised, and an astringent soon relieved the traumatic conjunctivitis. The case is interesting on account of the size of the fragment, its retention there so long a time without causing more worry, and the small amount of damage to the cornea and adjacent tissues.

**Iodin in Trachoma.**—Yet another treatment for trachoma. E. A. Nesnamoff,[3] of Charkow, after a three years' use of iodin for this affection, recommends it strongly. He employs it with either glycerin or petroleum ointment, and claims that every form of the disease is curable by its persistent use. Not only are the granulations absorbed and pannus cured, but even old scars in the lid are so much altered that they no longer injure the cornea. He uses the glycerin solutions if there is much secretion, but for the greater number of cases prefers the petroleum. He commences with one-half per cent. solution, and gradually works up to three or four per cent. Applications are made daily, the lids being held well open and thoroughly everted. He says iodin is soluble in the substances mentioned to the extent of about one and a half per cent. If a stronger solution is desired, it must be obtained by adding sufficient alcohol to the glycerin, or ether to the petroleum. Such solutions should be kept in well-stoppered bottles in the dark, and frequently renewed to keep up their strength.

**Operation for Entropion.**—By the way, talking of trachoma, with its resulting cicatrices and entropion, F. C. Hotz,[4] of Chicago, maintains that the prevalent idea, viz., that entropion is caused by shrinkage of the tarsal cartilage and cicatricial contraction of the conjunctiva, is wrong. He says, if closely studied, it is found that the anterior edge of the lid margin with the lashes is displaced long before the posterior edge shows any disturbance. He believes the dislocation of skin and lower bundles of the orbicularis is brought about by the oft-repeated spasms of that muscle. On this account the indication in operation is to draw the dislocated skin and muscle up on to the external surface of the tarsus, and fasten it there to prevent its slipping down. This is accomplished by an incision parallel to the lid margin, the removal of the muscular fibres over the upper border of the tarsus, and suturing the skin drawn up from the lid margin to the upper tarsal border. In some cases he finds it necessary to add an incision in the lid margin, which, being made to gape, is filled with a graft of skin or mucous membrane—usually of skin, cut from behind the ear.

**Hydrogen Dioxide in the Treatment of Marginal Blepharitis.**—Dr. Ayres,[5] of Cincinnati, finds that the hydrogen dioxide acts as an excellent application for softening the scabs so well known in this troublesome complaint. He first used it in its ordinary strength, but found that when a drop got on the conjunctiva it caused considerable pain. He then reduced it about half with water, and found that this strength gave equally good results, without any of the disadvantages of the stronger solution. He applies it by means of absorbent cotton rolled round a small piece of wood. This is dipped in the dioxide and rubbed along the lashes. The application should be kept up till the specific oxidising effect is seen on the crusts, as will be evidenced by the bubbles. He says, furthermore, the advantage of the dioxide is that it has a softening effect on the scabs, which water does not have. A white line of bubbles will be seen along the edge of the crusts, which seem to swell. Keep this up for some time, and the edges of the crusts will be seen to separate; then rub the lids dry with absorbent cotton, and the crusts will become partly detached. If they should prove very hard and dry more dioxide must be used, and more time given it to soak in. Then, with a probe or scoop, they can be lifted off without causing any pain. The dioxide, besides its mechanical use, is a great antiseptic and disinfectant, while it is neither toxic nor corrosive, but merely acts by oxidation, and does its work quietly and painlessly converting pus into inert matter, while it cleanses diseased surfaces as thoroughly as would the stronger preparation.

**Mydriatics in Glaucoma.**—Jackson[6] points out that mydriatics, instead of doing harm in glaucoma, do good in certain cases, and says, "But while mydriatics show a liability to increase the obstruction of the filtration angle, and thus to raise the tension of eyes already glaucomatous or upon the verge of glaucoma, they probably have no more general tendency to cause glaucoma, and they may by reducing intraocular congestion or in other ways exercise a beneficial influence upon glaucomatous eyes that they cannot injure by dilating the pupil." Furthermore, the liability to do harm in glaucoma appears to be shared by all the mydriatics. As each new drug of this class has been introduced the hope has been expressed, and sometimes apparently sustained by the history of the earlier trials, that it would not have this influence in glaucoma, but subsequently cases of this kind of harm from its use have been reported. In practice, however, the two mydriatics, homatropin and cocain, may be regarded as free from serious danger of causing permanent injury to the patient, since their effect upon the iris can be promptly overcome by eserine. As he found experimentally, eserine is capable of neutralising the effect of six times its weight of homatropin upon the iris and ciliary muscle. This is in strong contrast to the influence of atropin, which overcomes five, or of duboisin, which overcomes the effect of twelve times its weight of eserine.

With cocain it requires but one twenty-fifth of its weight of eserine to neutralise its action on the pupil; even one-fourth of its weight of pilocarpin is sufficient to do the same thing. And aside from its action on the iris in dilating the pupil, the tendency of cocain is to diminish the intraocular pressure by causing constriction of the blood vessels.

In any case in which the pupil is firmly bound down by adhesions, or is otherwise so fixed that mydriatics cannot cause thickening of the iris opposite Fontana's space at the angle of the anterior chamber, especially if the application of eserine aggravates the symptoms, it is justifiable to use atropin or some other mydriatic; and in a small proportion of cases such applications will be of marked benefit. In the great majority of eyes, a mydriatic cannot cause glaucoma at any time of life; while, on the other hand, a few patients are affected with the disease even from childhood. The danger is best guarded against by bearing in mind the symptoms of glaucoma, and always looking for them before ordering a mydriatic, especially by a careful opthalmoscopic examination.

[1] Therapist, April 15, 1898. [2] International Med. Jour., Mar., 1898. [3] Amer. Jour. Med. Sci., Mar., 1898. [4] Ibid. [5] Therapeutic Gazette, Feb., 1898. [6] Amer. Jour. Med. Sci., April, 1898.

